# Useful Formulas

**Published:** 1896-12

**Authors:** 


### USEFUL FORMULAS.

For irritable eczematous eruptions the following prescription has been found very useful in canine practice by Dr. Cecil French:

| | |
|---|---|
| ℞.—Sulphuris sublimati | ℥iv. |
| Olei picis liquidæ | ℥viij. |
| Camphoræ | ʒj. |
| Saponis viridis | ℥iv. |
| Alcohol | ℥viij. |
| Aquæ calcis | Ojss.—M. |

For mange (sarcoptic) add oil of cade ʒiv to the above.

For severely inflammatory, irritative, rheumatic eczemas, which alternate with true rheumatoid lameness, prescription No. 2 is preferable when accompanied by proper internal treatment:

No. 2.

| | |
|---|---|
| ℞.—Acid. boric. | ʒj. |
| Acid. carbolic. | ʒij. |
| Glycerin.<br>Ext. hamamel. fl. | āā Oj.—M. |

Add to the above ʒiij to ʒv menthol dissolved in sufficient alcohol.

*For Eczema in Dog (Dr. Gill).*

| | | |
|---|---|---|
| Talcum, Amyl (starch) | āā | 100 parts. |
| Glycer. | | 40 " |
| Aq. plumb. | | 100 " |
| Sol. acid. boric. (2 : 100) | | 100 " |

*Formula of Periostine (Sweat Blister).*

| | |
|---|---|
| Iodine | ʒx. |
| Kali iodid. | ʒiv. |
| Camphor | ʒij. |
| Wood alcohol | ℥viij. |

Apply with camel's-hair brush.

*For Distemper.*

| | | |
|---|---|---|
| Magn. sulphas | | ℥ij. |
| Kali chloras | | ʒij. |
| Liq. ammon. acet. | | ℥iv. |
| Spts. ether. nitros. | | ℥iss. |
| Fl. ext. bellad. | | ʒj. |
| Oxymel | | ℥iv. |
| Aqua | ad | Oj.—M. |

Sig.—If high temperature, give the above three times a day, reducing dose as fever subsides; later tonics or stimulants may be added.

*Solution for Preserving Brain and Upper Cord.*

| | |
|---|---|
| Corrosive sublimate | 60 parts. |
| Salt | 120 " |
| Acet. acid | 120 " |
| Water | 2000 " |

*Best Preservative Fluids.*

Saturated solution of *pure* corrosive sublimate in water.

Formaldehyde, 2 per cent. solution in water. (Formalin is a 40 per cent. solution.)

Alcohol 95 per cent.

Whiskey and brandy varies from 40 to 60 per cent.; ordinary alcohol is about 50 per cent. Nothing less than 95 per cent. is worth using.

Sublimate is the cheapest; formaldehyde keeps the color of the specimen best.

No other preservative fluid worth any consideration. It is not worth while to attempt to save specimens which have become putrid. Enough fluid should be added to cover a specimen completely.

---

IMPORTANT veterinary meetings: Schuylkill Valley, at Pottsville, Dec. 16, 1896; Niagara and Orleans County, at Niagara Falls, January, 1897; Virginia State Association, at Staunton, Jan. 8, 1897; Colorado State Association, at Denver, Jan. 5, 1897; Chicago Veterinary Society, Dec. 10, 1897.

